# Expired patents: An opportunity for higher education institutions

**DOI:** 10.3389/frma.2023.1115457

**Published:** 2023-03-17

**Authors:** Mhlambululi Mafu

**Affiliations:** Department of Physics, Case Western Reserve University, Cleveland, OH, United States

**Keywords:** expired patents, intellectual property, public domain, patent database, sustainable development, patent information

## Abstract

Patent protection typically lasts about 20 years from the filing date and is in exchange for sufficiently disclosing the invention. The disclosure aims to enrich technical knowledge globally, promote creativity and technological innovation and contribute to sustainable socio-economic development. After this protection period, the patent expires, and in principle, any person may begin practicing the specific subject matter previously protected by the patent. Since the invention originally met all the patentability requirements, it was disclosed sufficiently to stimulate further innovation by others through a thorough understanding of existing developments in the patent literature. Thus, in addition to scholarly research articles, this makes patents potentially valuable sources of technical information in research and academia, unlocking new technology opportunities. We use the exploratory research method to study a potentially genuine and vital research stream that uncovers the overlooked yet valuable scientific and technical information sources that higher education institutions could utilize to complement academic research articles. This work establishes a necessary research agenda that critically challenges researchers to tap into the immediately available and promising technology opportunities presented by patents in the public domain. Using case studies to gain in-depth, multi-faceted explorations about the impact of these patents, we find that technologies contained in expired patents, abandoned patents, and technologies not protected by IPRs, resulting in improved research quality and increased collaboration with industry, if adequately exploited and integrated with other technologies. Moreover, this could lead to increased academic patenting and commercialization with support from the university's Technology Transfer Office.

## 1. Introduction

Intellectual property (IP) refers to the creations of human ingenuity, creativity, and inventiveness (WIPO, 2004; Higgins and Graham, 2009; Bently and Sherman, 2014; Adams, 2019). Essentially, IP is divided into two categories, i.e., industrial property (i.e., patents, industrial designs, and geographical indications) and copyright (i.e., literary works, films, music, artistic works, and architectural design) (WIPO, 2004; Bently and Sherman, 2014). One of the essential phenomena of IP is that it has a legal character and substance. Hence, instead of just basic knowledge and having a creative advantage, it provides a defined scope of private property, the system of ownership of rights, and enforceable legal monopoly rights (Evenson, 2019). Thus, it is critical to establish an appropriate balance between the interests of IP owners and broader society though challenging in some contexts. An example of intellectual property right (IPR) is a patent. A patent is a set of exclusive rights granted by a sovereign State or a government office upon application by an inventor or assignee for a limited period in exchange for detailed public disclosure of an invention. The disclosure allows the dissemination of knowledge for others to recreate and build on the patented innovation, thus stimulating further technological innovation. An invention refers to a solution to a specific technical problem or a product or process (Bently and Sherman, 2014). Notably, the invention must belong to a patentable subject matter and be novel, exhibit a sufficient inventive step, be industrially applicable, and sufficiently disclosed in the application (Bently and Sherman, 2014; Halt et al., 2019). The grant of a patent provides the holder with the right to exclude others to; make, use, sell or offer to sell or import the patented invention or through the outright sell of a patent during its lifetime. This means anyone without authority from the patent owner will infringe on the invention, and the owner can sue for damages if they carry out any of those rights (Evenson, 2019; Dent, 2021). Moreover, these rights may be dealt with commercially by any party, including university researchers and academics, as with any other private property, for example, be sold off or licensed on an ongoing royalty basis.

In IP, the right to exclude others is of fundamental importance because it allows the inventor to derive material benefits as a reward for the work, intellectual effort, and compensation for expenses incurred during research and experimentation that culminated in the invention (Shavell and Van Ypersele, 2001; Cammarano et al., 2017; Yu, 2017; Battersby, 2019). However, allowing patent holders to exclude others from exploiting the invention could prevent further technological development in that field. Therefore, the patent system provides a conducive environment where creativity and technological innovation can flourish for the public benefit and simultaneously offer ways to control it (Bently and Sherman, 2014; Adams, 2019). As a result, for patents to encourage innovation without holding it back or allow others who want to improve on the underlying invention, patents must, at some point, expire to balance these competing concerns. Upon expiry, the patent enters the public domain, and holders lose all their patent rights and cannot prevent others from exploiting their invention (Bessen and Meurer, 2009). Once in the public domain, the licensing agreements for collecting royalties from the patent become unenforceable. Moreover, an invention falls into the public domain and could be available freely for use if it becomes abandoned. Abandoned patents refer to those allowed to lapse by their owners before the end of the entire available period of patent protection. According to the US system, this lapsing results from an owner's failure to pay maintenance fees due 3.5 years, 7.5 years, and 11.5 years after the grant. Therefore, the failure to pay fees causes the patent to lapse. However, suppose the patent owner unintentionally or unavoidably missed paying the maintenance fee. Then, the patent can be reinstated by filing adequate petitions that a Patent Office regards satisfactory to show that it was unintentional or unavoidable (Ochoa, 2002). Most significantly, mere expiration or abandonment does not automatically mean anyone can begin to practice the invention freely because a single patent application could result in numerous patents (Halt et al., 2019). The invention may also be covered by an industrial design or a combination of a patent and industrial design. Also, certain features of the invention may be protected by trademarks or copyright. Additionally, the invention may have utility or ornamental design improvements, which may be entitled to further patent or industrial design protection. Hence, because one patent has been identified and established that it has expired does not mean it is available for exploitation. To exploit the invention, a user must receive a freedom-to-operate analysis or any other commentary from a lawyer indicating that what they intend to do is legal and does not violate any other IP rights.

The patent system grants property rights to inventors in exchange for disclosing their inventions to the benefit of the public. The disclosure includes technical information, legal information, business information, policy-relevant information, and business information. The information about inventions disclosed in patent documents provides a rich source of sufficient and relevant technical knowledge. Moreover, it offers progressive ideas and solutions to technical problems in a particular technology area. This leads to inventions with improved attributes to impact society and brings positive changes. The biggest beneficiaries are the public, who benefit from promoting a more innovative culture in the short and long term by having guaranteed access to the technology once the patent falls into the public domain. Considering that a patent usually expires after 20 years, at the end of the expiry period, some of the disclosed information or knowledge will no longer be novel, especially in fast-changing technological fields. Therefore, academic researchers at HEIs or industries can only benefit if they have access to live patents since they usually work at the forefront of most technologies. However, other researchers interested in reverse engineering to solve community problems can fully utilize even older or expired patents. Notably, researchers at HEIs can exploit live patents by leveraging the principle of territoriality. While the rights granted by a patent are territorial, the disclosure is worldwide. However, this does not imply a worldwide patent; it only means that the information contained in the patent document is available worldwide. Most significantly, one of the conditions for granting a patent is that there must be sufficient disclosure, such that any person skilled in the art to which the invention pertains must fully understand the invention and can easily carry out the invention. Therefore, any interested party or entities at HEIs in jurisdictions where the patent was not designated upon grant, can exploit this principle by learning and building the exact copy of the patented invention and bringing it to the market based on this knowledge. On the other hand, the researchers at these HEIs could directly practice the invention instead of attempting to improve or reverse engineer it. Thus, this could save the institutions time and financial resources, for instance, going through project proposals, prototyping, and testing. Subsequently, this may lead to successful projects leading to products or services and start-ups and assist in solving long-standing community challenges. This demonstrates that information about live or expired patents significantly encourages technological innovation, research, and development (Blackman, 1995; Bregonje, 2005; Price and Nicholson, 2016). Particularly, utilizing patent information prevents duplication of research activities, lessens the utilization of material and financial resources, raises the standard of research and planning activities to reach the level of the most advanced technical solutions, and avoids infringing on someone else's IPRs (Bregonje, 2005; Bently and Sherman, 2014). Patent information is found in various patent databases. Often, it represents the only source of information about some technical solutions, keeping in mind that it is the first publication and sometimes the only one due to the novelty requirement (Blackman, 1995; De Weck, 2022). The databases have been designed to make the patent landscape more transparent and include Google Patents, Patentscope, USPTO, Espacenet, The Lens, and United States Patents Quarterly. Using a patent title, inventor's name, patent number, current patent owner, and technological field, researchers can use these databases to systematically retrieve data at a large scale and check if a patent is still in force or has entered the public domain (Blackman, 1995; Maravilhas-Lopes, 2020a). Moreover, a published patent contains an anticipated date (i.e., the longest likely date that a patent can last) written on its front page, which can provide information to check if the patent is still active. This allows patent and non-experts to effortlessly access their field of interest and gain valuable information to improve the invention or develop a new one. This also allows them to gain insight into sustainable innovation in distinct fields across the globe. Therefore, universities can use these opportunities for improved decision-making and technology commercialization strategies, including exposing cost-saving opportunities. While patent documents are usually presented in a standard format, researchers may liaise with competent staff from their Technology Transfer Offices (TTOs) to assist them in performing a patent search to leverage patent intelligence. A patent document usually comprises, amongst other parts: (a) Bibliographic data-on the front page is printed bibliographic data, which includes title and abstract, but also dates, names, and classifications, and (b) Text: title, abstract, description and claims-collectively the full text. To perform a search, a researcher can use an applicant or inventor's name, patent classification, or keywords and phrases. Patent information search is essential to:

i. Check if a similar patent to your invention already exists
ii. track the progress of a published patent application
iii. See if patents are available to license
iv. Search patent documents to determine if a planned innovation project might infringe on another firm's patents
v. Check if other firms are infringing their own patents
vi. Challenge a competitor's patent application, and
vii. Compile information on the prior art as part of their own patent applications.

Several types of searches exist. These include pre-application searches, state-of-the-art searches, patentability or validity searches, infringement searches, and technological activity searches.

While various lead to a patented invention entering the public domain, the most critical thing is that the invention had once received patent protection. Most significantly, a significant number of granted patents promise to positively impact the communities, thus leading to sustainable socio-economic development (Maravilhas-Lopes, 2020b). This means the application contained sufficient disclosure for enriching technical knowledge globally, promoting further creativity and innovation; hence a patent was granted. Thus, this sufficient disclosure affords patents to contain a comprehensive technical and business information source, making them a potentially valuable intangible asset for researchers to reproduce the invention (Brown, 2006; Crowe et al., 2011). Therefore, entering the public domain does not invalidate the patent information contained in the document. Furthermore, considering that some of the inventions may have received industry funding and were intuitively more likely to result in commercial success since they were often the result of a request or need from the private sector, thus this suggests their patent value. Thus, instead of entirely depending on scholarly research articles, we recommend that HEIs could gain valuable information by exploiting patent information in expired patents or those which have entered the public domain. As a result, this article addresses the following research questions.

i. What is the rationale for using expired patents or patents that have fallen into the public domain as scientific and technical information sources for researchers and academics at HEIs?
ii. How would HEIs benefit from information on expired patents or live patents to bring the change leading to technological development?

Except for this introduction, we arrange this work according to the following. Section 2 reviews the literature related to this work, while section 3 provides the methodology. Section 4 examines the rationale of patent information and how academics and researchers at HEIs can derive value from expired patents or patents in the public domain. Moreover, we discuss relevant case studies and suggest how HEIs can strategically use patent information for their benefit. In section 5, we discuss the challenges and opportunities presented by expired patents or patents in the public domain, and lastly, in section 6, we provide the conclusion and recommendations.

## 2. Literature review

Patents form one of the most common IPRs and provide a limited monopoly granted in return for the disclosure of technical information (Bently and Sherman, 2014). The disclosure enables a person with ordinary skill in the relevant art to repeat the invention based on the description in the published patent document. However, to create a sustainable balance between the inventors' interests and those of society, a patent will enter the public domain at the end of this monopoly or patent protection period (Maravilhas-Lopes, 2020a). Consequently, the right to exclude others from exploiting the invention ceases, and competitors may freely practice the previously patented invention. On the other hand, this may lead to pricing competition as the invention reaches the market with goods probably made during the patent lifetime (Lemley, 2008; Vishnubhakat, 2014). A case in point was the modification of the South African Patents Act, which now allows third parties to use patented inventions for non-commercial research and development purposes while the patent is still in force. Thus, this enables users to enter the market immediately upon the expiry of the patent (Wolson, 2007).

The “public domain” is the status of an invention, creative work, commercial symbol, or any other creation not protected by any form of IP (Kop, 2019). This means all the rights previously held by the owner (or licensees) cease to exist. As a result, any member of the public can use the technology described in the patent without a licensing fee or fear of a lawsuit for infringement (Kim and Lee, 2017). Notably, at the same time, technology falls into the public domain because IPR protection was never sought in the first place. Most significantly, entering the public domain does not invalidate or render the information contained in the patent document obsolete or not useful. The technology can still find various applications if incorporated with other technologies and birth excellent opportunities in other fields (Clancy, 2018). These opportunities could be in a similar technological area or a different one. For instance, the electronics industry is quite volatile, which means many technologies are being realized daily. Therefore, numerous patents are being granted in this field. Accordingly, it is expected that out of these patents, numerous expire and fall into the public domain; hence opportunities for expired patents are expected to be high in this field. However, this might differ from sectors such as biotechnology or pharmaceuticals, where expired patents may still be protected through patent families. Remarkably, the use of patents as sources of information can assist in expanding sources of innovation leading to new technical opportunities (Dratler and McJohn, 2022). Moreover, several authors have discussed the possibility of realizing new technological opportunities by integrating existing technologies (Palmberg, 2004). These technologies include inventions mainly from expired patents. Furthermore, the authors in Palmberg (2004) analyzed the value of expired patents by comparing them with valid patents. They found that expired patents may hold as much value as valid patents depending on the approach or technology involved. Upon utilizing some commonly used criteria for investigating patent values, authors found that expired patents appeared to possess the same features as unexpired ones. This means if expired patents are exploited appropriately, they could be excellent references for developing future technologies and sources of innovation. Particularly, a patent enters the public domain when:

i. The patent has reached its legally prescribed protection period of about 20 years. The life of a patent starts when the application is filed. On average, the Patent Office takes about 2 years to approve an application, so inventors have even less time to use a patent than its maximum life. When the life of a patent runs out, it expires automatically and falls into the public domain,
ii. The patent holder goes out of business and abandons the patent by not paying the maintenance fees or neglecting to pay the maintenance fees on time or within the grace period provided. Unfortunately, the maintenance fees usually increase over time to encourage the patent owner to relinquish their rights to the public. The maintenance fees are due at 3.5 years, 7.5 years, and 11.5 years after the grant. The late payment can be made within 6 months of the due date. Unfortunately, if the maintenance fee is not paid up, the patent expires and gets into the public domain,
iii. The patent holder has been able to monetize the invention but concludes that they have extracted all returns associated with the invention even before the end of the patent term, and the patent holder reaches a conclusion that they will not be able to recoup all the costs that they expended in the invention or that the patent is not worth the cost of maintenance,
iv. After it has been invalidated by a Court of law, i.e., after its grant, it is found to be anticipated by material belonging to the public domain and must not have been granted. Therefore, the Court will review the Patent Office's decision to grant the patent, and if it finds a mistake in granting the patent, then the patent is invalidated, and
v. The USTPO has the authority to review its decision to grant a patent and may revoke it, causing the holder to lose all their rights and the invention to enter the public domain.

Recently, Park et al. (2021) collected expired patents from major domestic universities and compared the quality characteristics of the expired and live patents for their long-term use (Park et al., 2021). Remarkably, the authors found essential patents among expired patents. Using machine learning models, they predicted the use of unexpired and expired patents and contributed to setting goals for research results from technical collaboration between industry and universities. Mainly, they identified firms and fields of application to which the patents can be utilized, such as in university-industry collaborations to increase the efficiency of technology transfer and commercialization (Mafu, 2023). The university-industry partnerships link fundamental scientific research and patented industrial technology, which were long established (Narin et al., 1995; Eisenberg, 2000; Meyer, 2000). Following these collaborations was an increase in knowledge transfer, and substantial growth in academic patents leading to the development of numerous methods, products, and technologies (Sanberg et al., 2014; Freitas and Verspagen, 2017; Arant et al., 2019; Kang, 2020; Ferreira et al., 2022). Moreover, Price and Nicholson (2016) studied the relationship between expired patents, trade secrets, and stymied competition. The authors examined the practice of some patent holders in manipulating the complementarity and substitutive relationship between patents and trade secrets to maintain their monopoly and meaningful competition even after the expiration of the patent exclusivity. Consequently, they proposed a concept of economic enablement, where patentees may be responsible for enabling the bare technical invention to be disclosed in a patent and the minimum information necessary to exploit the patented invention commercially. Notably, Yun et al. (2021) investigated technology opportunities presented by expired patents, especially within the target technological field named as a promising technology, and opportunities from other technical fields called convergency technology. They found that patents with high quality and low implementation risk present technology opportunities and imply that expired patents can be as crucial as valid patents as sources of new technology opportunities. Therefore, with adequate guidance for expired patents, their proposed approach is anticipated to reduce the costs and risks of adopting them (Yun et al., 2021). Furthermore, Gruner (2021) studied the impact of abandoned patents in later technology development. The authors discovered that citations for unabandoned patents were two times higher than those of the inventions described in abandoned patents. Broadly, this demonstrates abandoned patents influence later technology development though less than the unabandoned patents. Since abandoned patents are freely available, they may still present opportunities for educational purposes for HEIs.

In contrast, expired patents have higher citations than unexpired ones. This could suggest a menace of patent enforcement when a patent is still in force is influential in quashing technology development. Thus, the level of innovation related to critical advances increases when the legal danger of patent enforcement is reduced on expiration. Moreover, this demonstrates that expired patents significantly influence sustainable technology development, and HEIs can leverage them to construct non-obvious improvements to past technology designs and even realize new patents or patent portfolios, leading to revenue generation through licensing (Sanberg and McDevitt, 2013). This provides an alternative or an addition to more conventional sources of academic funding through research grants. The benefits of patents and commercialization lead universities to access unrestricted funds for further institutional investment, sustaining high scholarship levels, student success, public benefit, and economic development (Sanberg and McDevitt, 2013; Drozdoff and Fairbairn, 2015).

## 3. Methodology

We use the exploratory research method to investigate how academic researchers at HEIs can leverage patent documents in the public domain to complement scholarly research articles to derive state-of-the-art research information. The exploratory methodological approach is suitable for examining research questions or tackling new problems on which little or no previous research has been done or has previously been extensively studied (Brown, 2006; Saunders and Lewis, 2012). Although it is conducted to determine the nature of the problem, exploratory research is not intended to provide conclusive evidence but assist in a better understanding of the problem. This approach is appropriate for our study since we aim to build a future research agenda for future studies. Considering that little information is known about this subject, we leverage several methods to construct the research design, collect data and choose subjects. Mainly, we use case studies and literature reviews to collect our secondary data. The case study approach permits in-depth, multi-faceted explorations of complex issues in their real-world settings (Crowe et al., 2011). We selected these cases and related literature not because they represent other cases but due to their uniqueness and potential to address our objectives. The data gathered is qualitative data. We use the case study as a research strategy since it forms a valuable tool for the preliminary, exploratory stage of research projects and forms a basis for developing the ‘more structured' tools necessary for future surveys and experiments (Rowley, 2002; Crowe et al., 2011). Furthermore, case studies assist answer “How?”, “What?” and “Why?” questions. In this role, they can be used for exploratory, descriptive, or explanatory research (Yin, 1994; Gomm et al., 2000). At HEIs, our most suitable candidates are TTOs, technopreneurs, innovators, developers, and academics. At HEIs, these candidates often face unknown situations or future events, such as adopting new products, assessing new technology impacts, or other disruptive events. Typically, when investigating such a unique and complex situation, the researcher must base their assessment on known models or closely related secondary sources from where they may deduce information to make a forecast. Notably, to make a forecast, case studies provide an excellent starting point, among other techniques (Steinert, 2009), to discuss the potential of patents or technologies in the public domain to address our objectives. We extensively searched for a broad scope of sources consisting of scholarly articles, patent documents, policy documents, and legal documents. Also, we peer-reviewed literary sources and various databases, mainly Google Scholar, preliminary documents, commentaries, online articles, patent databases, and ProQuest (Interdisciplinary). The search covered the years from all the years until 2023. We analyzed the titles, abstracts, and reference lists for articles indexed in these electronic databases. We accessed these sources through the Case Western Reserve University Library website. To search, we used keywords such as “expired patents,” “public domain,” “intellectual property,” “patent databases,” “patentability,” “higher education institutions,” and “patent infringement.” As inclusion criteria, we chose sources and articles, particularly case studies that contained these keywords or were relevant to our research topic, using our prior knowledge and understanding. We excluded duplicated articles, did not report on our research topic, or did not include our keywords. Furthermore, we excluded pre-prints that were not peer-reviewed, covered different topics, or had insufficient data or unavailability of full text or non-English articles. As part of the quality assessment and based on our knowledge about this subject, we validated the consistency of information from our sources by comparing viewpoints from different authors. Compared to other methods, one of the great strengths of case studies is that evidence can be collected from multiple sources. Finally, using relevant authoritative sources, especially by leading authors or highly cited sources, we assembled our research into the current article using the triangulation method. The triangulation technique uses evidence from different sources to corroborate the same fact or finding (Rowley, 2002). This technique establishes the rationale for using expired patents or patents available in the public domain as sources of scientific and technical information and scholarly articles by researchers at HEIs. As with other methods, the limitations associated with this methodological approach impacted the results of this study. For example, except for the mentioned sources, we are unaware of other peer-reviewed articles or work with related objectives, restricting our ability to collect and analyze data. Moreover, this approach is influenced by the researcher's subjectivity and external validity. However, though inherently challenging, we believe that our study approach would produce more powerful insights if we had numerous sources of relevant information.

## 4. Patent information

Patent information is a piece of legal and technical information found in patent documents and is published by the Patent Office (WIPO, 2004; Paranjpe, 2012; Maravilhas, 2015). The information consists of patent applications, granted patents, and expired patents. Also, it contains business-relevant information from patent claims defining the patent's scope and legal status and public policy-relevant information from analyzing filing trends (Lubis and Baiti, 2018). Therefore, in addition to other information sources, the TTOs at HEIs could leverage this information when assessing the commercial potential and success of the inventions of its researchers. Also, they could use these trends to study areas where it could encourage innovation and engage in entrepreneurship to bring a commercial product to the market. Moreover, researchers can use this information to track other competing universities or start-ups' patenting activities (Kim et al., 2008; Martin and Mykytyn, 2010). This will allow researchers to understand progress and identify gaps in technology development. As a result, HEIs could use this information to avoid unnecessary investment or potential risks, support the university's technological innovation development, and inform a strategy for technology investment and policy (Lee et al., 2009; Yoon and Kim, 2012). Notably, about 80% of technology information is directed at solving technical problems, most of which is first published in patent documents (Bloom et al., 2019). Besides containing information often not divulged in any other form of literature, patent documents also provide examples of an invention's industrial applicability and cover practically every technology field. This is critical to technopreneurs and researchers from science and technology universities.

Using patent analytics (patent landscaping or patent mapping), a qualitative or quantitative analysis of patents filed by other inventors to gather intelligence on technology, market trends, competitors, and new commercial opportunities. This is critical in strategic planning and informed decision-making. Moreover, researchers can employ visualization methods to data generated from patent information to gain comprehensive insights (WIPO, 2004; Kim et al., 2008; Yoon and Kim, 2012). These insights contain intelligence to assist in exploiting knowledge, improving an entity's productivity, and increasing product differentiation, thus providing a basis for a longer-lived competitive advantage (Grant et al., 2014; Bloom et al., 2019). Particularly, patent information is one of the most critical indicators based on both a resource- and knowledge-based view. It successfully identifies technology opportunities researchers can leverage to gain a competitive advantage in developing highly innovative products or address community technical challenges.

Furthermore, patent information provides a critical resource for value creation and innovative technological subjects, capabilities, and knowledge (Vyukhin et al., 2016). Accordingly, inventors at HEIs can use this information to track the dynamic evolution trends of different creative technical topics in a target technology and identify growth opportunities in emerging markets (Zekos, 2004; Maksimova, 2014). Researchers at HEIs could formulate sophisticated patent strategies to obtain a competitive advantage for technological innovation, which could lead to spin-offs or start-ups. Since patents are granted before a patented product is introduced, patent information provides updated information relevant to the business (based on reference data identifying the inventor, filing date, and country of origin) (Hunt et al., 2012). Therefore, TTOs at HEIs can use this information for their strategic planning purposes (such as competitor monitoring, analyze technology landscapes, technology assessment, determining future-oriented technologies and R&D portfolio management) to identify and assess potential sources for the external generation of technological knowledge, primarily through licensing and human resource management. Moreover, this allows TTOs to provide researchers with insights about technologies that could make an impact or are less relevant. This demonstrates that patent information forms a core ingredient in HEI's knowledge management system (Ernst, 1998; Fabry et al., 2006).

### 4.1. Deriving value from expired patents or patents in the public domain

Expired patents, live patents, or IPRs whose protection was never sought in the first place and became available in the public domain present several potentially valuable and opportunities for HEIs. For instance, researchers and academics at HEIs can learn the state-of-the-art in a particular field, what inventors have developed and disclosed in their patents and patent applications. In addition, this may offer opportunities to know what has not been claimed and disclosed in the patents and applications. Accordingly, based on patent information from expired patents or patents in the public domain, the researchers can skip what has been claimed or use new processes that were not available in the patent (e.g., additive manufacturing) to advance state-of-the-art with their new patent application, keeping in mind that their patent application will have to survive the prosecution process. Therefore, with assistance from a TTO, researchers at HEIs may know where and how to proceed with their planned invention. Furthermore, this information could provide HEI with some intelligence about possible competitors through licensing information accessed from the Patent Office. Alternatively, if the technology is interesting, the HEI can pursue a licensing agreement with a party holding a valid patent instead of investing in expensive and time-consuming research and development (Elfenbein, 2007).

Moreover, knowing about patents in the public domain assist in avoiding duplicating research and development effort. Most significantly, it prevents researchers from potentially infringing other inventors' patents, as some of them could belong to some patent families still in force. Thus, this will save the number of litigation expenses and compensation for damages. In addition, based on this information, researchers, with the assistance from TTOs, can analyze the flow of technology from elementary technologies along with the expansion of those technologies, the trend of technological change, the lifestyle of technology, problems and solutions in the development of a particular technology, competitors' technologies, and solutions to cope with possible issues. In addition, knowing the lifestyle of technology makes it possible to judge the timing of development policy and focus on specific development themes. Notably, identifying critical trends in specific technical fields of public interest, like pharmaceuticals or the environment, will provide a foundation for research activities in which HEIs could focus, prioritize research funding or even develop human capital. For instance, patent analysis can help plan the human resources of a university. Suppose a university has a small number of highly prolific inventors who are driving technological development. On the other hand, it has a much larger number of researchers producing only one or two patents; patent analysis, such as a co-inventor brain map, can show the essential inventors that are vitally important for the future of the university. Thus, such brain maps can identify star inventors within the HEI and critical inventors in other universities, a valuable analysis of headhunting, and develop effective collaborations with other universities or industries.

Falling into the public domain does not invalidate that patent information is still critical for forecasting emerging technologies used in the patent. Based on the patent description and claims, academics and researchers could map creative activities and technological changes, particularly in the patent industry. Most significantly, depending on the time the patent fell into the public domain, there is a legal requirement that the invention for which protection sought must be novel. This means the invention must not have been disclosed anywhere in the world. This suggests that the invention contained in the patent literature is being described for the first time and, in most cases, nowhere else. Omitting this valuable information source means missing a large proportion of technical descriptions of novel research and technological innovation. Thus, HEIs could use this novel information to uncover the technological trends and areas that certain firms or industries are working on. This could allow HEIs to efficiently identify, monitor, and predict technology trends. Moreover, they can use this information to assess opportunities for future collaborations with industry or patent owners.

Leveraging patent disclosure could provide HEIs with a good starting point to improve the quality and impact of their scientific publications. For example, Breschi et al. (2006), Andries and Faems (2013) demonstrates that professors involved in at least one patent with the European Patent Office (EPO) publish more and even higher quality papers than their colleagues with no patents. It also highlights that they increase their productivity after patenting. Furthermore, studies show that most researchers, especially in biotechnology, appear to have outstanding research records after their involvement in patenting (Zucker and Darby, 1996; Agostini et al., 2015). This is also confirmed by studies in Azoulay et al. (2009), where they found the number of patents owned by scientists positively related to subsequent publication rates demonstrating the importance of patent information. Also, the publication behavior of academic inventors differs from their colleagues who are non-inventors working in similar research fields. Because academic inventors interact more with patent information, they publish significantly more in high-impact factor journals (Van Looy et al., 2006). These activities increase the chances for HEIs to solve real-world problems in a related area.

In 2005, Catherine Hettinger, the inventor of the ubiquitous finger spinner to assist kids with Attention Deficit Hyperactivity Disease (ADHD) and anxiety, struggled to raise the required maintenance fee of $400 after holding the patent for 8 years.^1^ Consequently, the patent for her genius invention lapsed and entered the public domain. A few years later, Hasbro, a giant toy manufacturing firm that had tested the finger spinner and rejected its original design started manufacturing improved designs. As a result, the fidget spinners have become the latest must-have toys and are being sold worldwide by large toy retailers.

Another interesting case is Tim Berners-Lee, the World Wide Web inventor who proposed the Web in 1989 and did not commercialize or patent his contributions to the Internet technologies he had developed.^2^ As a result, the World Wide Web has become an indispensable resource used by almost everyone globally, thus improving the quality of life and encouraging sustainable economic development. While the World Wide Web was never patented, its wide use demonstrates the revenue it could have drawn through the IP system. Most remarkably, on July 10, 1962, the United States Patent Office issued a patent to Nils Bohlin, a Swedish engineer, for a V-type three-point safety belt designed for road cars. The design provides a safety belt that, independent of the strength of the seat and its connection with the vehicle in an effective and physiologically favorable manner, maintains the upper and the lower part of the body of the strapped person against the action of substantially forwardly directed forces and which is easy to fasten and unfasten. While wearing a seat belt was not mandatory, in 1966, the federal law enacted a law to make them compulsory on every automobile. Notably, instead of licensing the patent to other automakers, Volvo decided that the invention was noteworthy because it had more value as a free life-saving tool than making corporate profits. Thus, they allowed every automaker to incorporate it into their vehicles. In 2009, Volvo estimated that the three-point seatbelt had saved more than 1 million people, though it keeps rising, displaying the value of this innovation.

On the other hand, in 2011, the hoverboard design inventor Shane Chen-who patented the original hoverboard, failed to profit from his invention as thousands of Chinese factories constantly manufacture cheap imitations.^3^ These imitations have flooded the market at about one-quarter of the cost of the original invention. This is a classic example where the patent system fails to protect the inventor's rights. While a patent gives its owner the legal right to exclude others from practicing the invention, for a limited period of years, in most countries, patent rights fall under private law, and the patent holder must sue someone infringing the patent to enforce his or her rights. Therefore, if the inventor has insufficient resources to pursue or sue infringers or companies that deal with or encourage counterfeits, they will never realize any profits from their invention.

Furthermore, a giant biotechnology seed company Monsanto was granted a patent in 1996 for the first-generation Roundup Ready One, a genetically engineered modified soybean designed to withstand herbicides (Edmisten, 2015). So, when the main patent that underpinned this technology, i.e., U.S. Patent Nos. 5,352,605 and RE39, 247 expired in 2011 and 2014; Monsanto no longer controlled one of its most significant inventions in the history of agriculture. As a result, some U.S. agricultural universities, including the University of Kansas, the University of Georgia, the University of Missouri, the University of Arkansas, and the Bay Farm Research Facility, began exploiting the technology to create GMO soybean varieties with more protection against specific local pests while maintaining high yields. Moreover, these university-developed seeds are generally half the original price, thus allowing farmers to cut input costs to save and replant the seeds, thus reducing farm expenses and lowering food prices, bringing public benefit. Moreover, these universities can also develop and begin to market their generic varieties and apply for a patent.

Since 2014, expired patents have transformed the Philippines and Papua New Guinea's local economies, where rural communities exploit old patents to develop their local economy (Expired Patents Transform Local Economy Future, 2022). For example, through the support of the Asia-Pacific Economic Cooperation (APEC), a remote village in Central Luzon, the Philippines, benefitted from the intervention of Korean IP experts to enhance the processing of ylang-ylang essential oils. Notably, the experts first analyzed the existing situation of the villages, followed by conducting prior art searches using the Korean IP database. Then, inspired by expired patents, they modified oil extractor boilers to maximize their capacity and developed a mobile facility to move between villages far away from the only existing extractor. Based on a similar approach, another APEC project successfully provided an irrigation system during the dry season in Pinu, Papua New Guinea. This intervention enables locals to feed their families during the period of hunger and sell the harvest and invest in healthcare and education. This demonstrates the impact of these recycled technologies where existing inventions are modified and adapted to local needs. Subsequently, this has raised the quality and standards of living in remote areas and boosted their economies through sustainable development.

According to L'Oreal 2013, mining expired competitive IP offers an excellent innovation strategy to create new IP or products immediately. L'Oreal has U.S. patents that cover marketed products or significant technology. For example, in 2013, it had 83 U.S. beauty-care patents expiring that formulators could take advantage of to innovate immediately. These patents are accessible through the FPO free patent search engine (freepatentsonline.com) (Useful Expiring US Patents, 2013).

### 4.2. Strategic use of patent information

Two primary ways of analyzing patent information are qualitative and quantitative analysis. The qualitative analysis method reviews the content of the individual technology in patent documents, while the quantitative method processes the statistical documentation of patents in each technology field (Expired Patents Transform Local Economy Future, 2022). Analyzing patent information provides business information regarding the target technology and its value before entering a licensing negotiation. When preparing to license the technology, analyzing patent information allows the inventor to determine whether the technology in question is in the public domain and the target market. It also assists TTOs at HEIs in finding out if the researcher will be sued for infringement and the possibility of the technology being overvalued or undervalued compared with other related or alternate technologies. Moreover, when negotiating a license or collaboration with an external entity such as a firm, patent analysis assists TTOs in addressing additional issues relating to whether the target's technology is as good as it claims to be. For example, the analysis will reveal if the company is reasonably priced, assist in identifying essential inventors, and if they will stay with the merged or acquired firm. This will be essential for spin-off companies at HEIs as they get into contractual agreements with external firms. Furthermore, while preparing to “license out” technology, patent information could clarify the identities of parties of prospective licensees in the marketplace and determine the value of the technology. For instance, when “cross-licensing,” patent analysis plays a pivotal role in comparing the patent portfolios of two or more firms and assists in deciding the amount to be paid by each party. This provides a picture of the life cycle of the target technology and key technologies in the field, which will help in the decision-making process. Thus, this information will benefit TTOs or researchers at HEIs when deciding which firms will be profitable to collaborate with or enter into an agreement.

Based on the patent analysis, researchers could monitor the activities of actual and potential competitors and identify niche opportunities for collaborative work. Furthermore, through the freedom-to-operate analysis or other legal commentaries, HEIs could use information from patent information for expired patents or those in the public domain to avoid possible infringement problems in case these patents belong to patent families or thickets. While other entities try to avoid infringing inventions due to the territorial nature of patent, the HEI's in jurisdictions where a patent was not designated can practice the subject matter described in the “live” patent documents. By leveraging patent information and appropriate patent analytics techniques, academic researchers can gain intelligence about relevant technical details and freely use patent documents to manufacture the product instead of reinventing the wheel. Moreover, the intelligence gained can be used by HEIs as input to forecast which technologies will be relevant for future development. Moreover, this helps to allocate technology development resources accordingly and reduces the risks and costs related to research and development. Thus, using expired patents, abandoned patents, and technologies in the public domain and leveraging the territoriality principle to solve existing societal challenges, better serve local communities in that area, and impact the world. Finally, while the patent system aims to protect IPRs, it creates a balance between the interest of the inventor and the public. Still, it must also be viewed as an essential resource for sharing technological ideas to result in technological innovations and sustainable economic development.

## 5. Challenges and opportunities

There needs to be more clarity about the goal of scientific research and the IP system (Zeebroeck et al., 2008). The main objective of science is generating knowledge and disseminating it. At the same time, a patent system enables disclosure, meaning it should be written so the invention can be reproduced. The disclosure of information in exchange for exclusive rights. This disclosure facilitates improvement in technology and allows improvement while building an essential database of technical knowledge. This knowledge is accessible to researchers or scientists at HEIs. It enables them to identify gaps and opportunities leading to developing second-generation products and processes to solve technical problems in unrelated fields. They may also use this specialized knowledge to identify areas where existing patents block new entrants in the technological field. As a result, a patent document can be seen both as a legal document and a scientific publication which may reach readers that very well be complementary to that of your favorite journal.

Moreover, there is a wrong belief that the two systems are too far apart or do not influence each other, i.e., they are orthogonal. For instance, some research academics at HEIs are concerned about the impact of patenting on the quality, quantity, or focus of their scientific research output (Hanel, 2006). Patenting has a positive influence on the quality and quantity of scientific publications. According to Meyer (2005), Zeebroeck et al. (2008), Arora et al. (2017), Bloom et al. (2019) academic research scientist typically produces more and increased quality research papers after patenting. Notably, patenting is generally associated with industrial funding, which positively affects the number of research papers. Moreover, some academics claim that patenting negatively influences the direction of their research focus or activities, resulting in reduced fundamental research that has proven to be unsubstantiated (Blossey, 2002; Magerman et al., 2015).

One of the conditions for granting a patent is the nondisclosure of the invention before filing a patent application. Therefore, some academic researchers believe that the patent system introduces delays in publishing and sharing their scientific results. Also, the increase in private research funding usually requires restrictions on the disclosure and timing of the publication outcomes. Finally, some academics believe they cannot publish a related article later if they obtain a patent. Furthermore, academics need to be made aware that this is a matter of balancing the two activities. Therefore, the procedure is that the researcher files their patent application, and before the application becomes public, they must submit their manuscript to a journal. In recent years, the US, China, and Europe have demonstrated a sharp increase in academic patenting, resulting in improved patent folios and fruitful university-industry collaborations (Markiewicz and DiMinin, 2004; Zeebroeck et al., 2008; Carraz, 2013; Sanberg and McDevitt, 2013). Some of the well-known academic patents which have generated significant licensing revenue include the Harvard College (Harvard Oncomouse) (Transgenic Non-Human Mammals, 1984), University of Edinburgh (Animal Transgenic Stem Cells) (Edinburgh, 2021), University of Bonn (Oliver Brüstle v. Greenpeace) (Straus, 2011), Michigan State University (Euthanasia Compositions) (Cohen and Boyer, 1980) and Stanford University (Cohen-Boyer Patent) (TBA, 2005). Moreover, some funders and investors require minimum IP protection, such as a patent application, before investing (Markiewicz and DiMinin, 2004; Carraz, 2013). Therefore, while these two systems have different goals, they complement each other.

In exchange for a patent, an applicant must disclose the information regarding the patent such that a skilled person in the art can reproduce the invention (Jaffe and Lerner, 2011). Unfortunately, patent documents are crafted by technological and legal minds. Finding many people with a good understanding of both fields may take time and effort. Thus, the language in which patent information (for instance, the claims) is presented may limit the number of people or discourage them from reverse-engineering or “inventing around” the technology. Notably, applicants value the “vague” language in which the claims are expressed because this allows manipulation at trial or during licensing negotiations (Burk and Lemley, 2009; Mullally, 2009; Jaffe and Lerner, 2011). In contrast, this enables them to be read narrowly, if necessary, to avoid prior art and broadly to ensnare third-party technologies. Therefore, the expertise required makes it impossible for competitors to reverse-engineer the technology effortlessly after its protection has expired. However, in some cases, competitors already working in the related technology after the protection period can reverse-engineer the technology, particularly pharmaceutical products and drugs. This means that this field does not easily allow new entrants except those already having sufficient knowledge about the technology or field. As a response, patent owners find a way to safeguard the information related to their inventions through trade secrets. This permits them to prevent competition until they invent or introduce another high-end product into the market. In such circumstances, patent owners benefit by using the patent system protection during the protection period and trade secret protection after the expiry of the protection period (Jaffe and Lerner, 2011). However, this exclusivity gained after patent expiry is based on secrecy and implies that competitors cannot free-ride since they do not know the necessary information about the invention. While the trade secret can persist indefinitely, requiring no registration, the setback is that no protection is granted to the inventor. If the invention is valuable enough, no further protection is granted to competitors who independently invent or reverse-engineer the invention contrary to patents. In the latter case, this is against the object of the IP system, where restricted monopolies are driving innovation. This phenomenon is unfair to the public because if there are limited monopolies, lower prices are not enjoyed from increased competition and decreased access, thus creating an unleveled ground for fair competition (Ayres and Parchomovsky, 2007). On the other hand, this implies the patentee did not disclose enough since people skilled in the art cannot reproduce the invention after its expiration. This non-disclosure regarding the best way to carry the invention will allow the inventor to continue having a competitive advantage and benefits over his competitors even after the patent expiry. However, like patents, a trade secret can lose economic value because the protected information has become obsolete, and the company will abandon the secret (Yun et al., 2021).

## 6. Conclusion

The study reveals that while patents may have expired or fallen into the public domain, they still present an invaluable opportunity for innovation and sustainable development and certainly one that can and should be pursued by researchers at HEIs. We demonstrated that HEIs are suitable candidates for these patents because they act as knowledge reservoirs and are at the forefront of innovation. Thus, in addition to scientific knowledge from research articles, HEIs could exploit the patent information to avoid unnecessary expenses in investigating what is already known, identify and evaluate opportunities for technology licensing and transfer, identify alternative technologies, and keep up-to-date with the latest technologies in the field of expertise. Moreover, patent documents provide ready solutions to technical problems and ideas for further innovation to positively impact society. Thus, patents in the public domain form valuable sources of new ideas, especially when they become available while they were still new or had not reached their maximum term. This usually occurs, for instance, after abandonment due to failure to pay renewal fees, or challenged before the Court of Law or live patents by exploiting the territoriality principle. We demonstrate from various sources and case studies that patent documents hold potential for commercialization and thus provide a genuine reason to study how they could effectively be utilized to realize new technology opportunities. Moreover, to corroborate this, it has been found that the citations for valuable expired patents are usually higher than those of unexpired ones suggesting their usefulness. While not all expired patents or patents in the public domain are valuable; however, their effectiveness or influence on subsequent technologies depends on their respective sector. However, in most instances, they are significantly less than their unexpired counterparts because the technology becomes obsolete. As discussed, the electronics industry gets numerous patents granted yearly, which typically means more patents expire annually and enter the public domain. Therefore, this sector, together with patents associated with the Internet of Things and Artificial Intelligence fields, which have short life spans, presents innumerable opportunities for patents in the public domain compared to patents granted in the pharmaceutical industry have long life spans. Notably, before the expired patents are adopted for use, researchers at HEIs must consult their TTOs to assess their value and the possibility of infringing live patents.

## Author contributions

The author confirms being the sole contributor of this work and has approved it for publication.

## Footnotes

^1^https://www.bgr.in/news/meet-catherine-hettinger-the-woman-who-invented-the-fidget-spinner-two-decades-ago-466045/ (accessed October 16, 2022).

^2^https://britishheritage.org/tim-berners-lee-the-world-wide-web (accessed October 16, 2022).

^3^https://zimhoverboard.com/who-invented-the-hoverboard/ (accessed October 16, 2022).
